# Creation and Validation of the First French Scale for Measuring Bore-Out in the Workplace

**DOI:** 10.3389/fpsyg.2021.697972

**Published:** 2021-07-23

**Authors:** Clément Poirier, Margaux Gelin, Moïra Mikolajczak

**Affiliations:** ^1^Laboratoire de Psychologie et d'Ergonomie Appliquée, Université de Paris, Paris, France; ^2^Research and Development Department of Moodwalk, Paris, France; ^3^Psychological Sciences Research Institute, Université Catholique de Louvain, Louvain-la-Neuve, Belgium

**Keywords:** bore-out, psychometry, scale validation, exhaustion at work, work bore-out scale

## Abstract

The aim of this study was to create and validate a scale of bore-out at work: a measure for bore-out that could be used in French-speaking workplaces. Bore-out is a recently defined phenomenon, and few studies have been conducted to evaluate it- particularly in a French context. We investigated the dimensional structure of bore-out in a sample of French-speaking workers by distributing an online survey. Exploratory and confirmatory factor analysis indicated four dimensions with high internal consistency. Through a measurement invariance analysis, a factorial structure was confirmed for men and women as well as for Gen-X and Gen-Y workers. Criterion validity was verified in regard to the relation between the scores on the WBOS dimensions and those obtained for self-esteem, depression, perceived self-efficacy, and perceived helplessness. The overall results of the analysis performed in this study show satisfactory psychometric qualities for the Work Bore-Out Scale (WBOS).

## Introduction

Over the last 40 years, the main phenomenon studied in the field of ill-being at work has been occupational burnout. Although beneficial, the academic focus on burnout tends to overshadow other reasons for unhappiness in the workplace such as harassment. Burnout equally tends to focus on the overwhelming nature of the workplace (e.g., having too much work or pressure) and unfortunately not as much attention is paid to how a lack of stimulation can be difficult for an employee. The role of boredom was raised almost a century ago (Wyatt, 1929), but has largely been neglected since then (Fisher, 1993). However, boredom at work is not to be neglected. In 2016, a survey of 23,236 French workers found that 41.1% of respondents suffered from boredom at work (Beque et al., 2019). In 2008, the phenomenon received attention with the invention of the term “bore-out” by Rothlin and Werder to describe the specific suffering stemming from under-stimulation and boredom at work.

### What Is Bore-Out?

According to Rothlin and Werder (2009), bore-out is composed of three components: *boredom, a lack of challenges*, and *a lack of professional interest*. According to their definition, *boredom* is a negative affective state that can stem from having nothing to do, but also from the specific type or content of employees' activities (e.g., meaningless, boring, or monotonous tasks). A *lack of challenges* is the feeling experienced by employees who perceive their tasks as below their abilities. A *lack of professional interest* appears when employees experience a loss of interest in their job, their company, or their career. As a consequence, employees may feel under-stimulated or unchallenged leading them to bore-out (Noriega, 2014).

More recently, Stock (2015) defined bore-out as “a negative psychological state of low work-related arousal manifested in three forms: a crisis of meaning at work, job boredom, and crisis of growth” (p. 574). These three dimensions are different from but not incompatible with Rothlin and Werder's definition. In both cases, *boredom* is a core symptom, a *lack of professional interest* can cause a *crisis of growth* (Abubakar, 2019) and a *lack of challenges* can lead to a *crisis of meaning* (Stock, 2015). These three dimensions represent a form of resource loss (Stock, 2015). According to the conservation of resources (COR) theory (Hobfoll, 1989, 2001), resources are “conditions, objects, energies, relationships and personal characteristics that are valued by the individual or that serve as a means for attainment of these objects” (Hobfoll, 1989, p. 516). The loss of these resources reduces employees' ability to invest their few remaining resources they do have at work. Individuals who suffer from bore-out typically enter a loss spiral (Abubakar, 2019).

Regarding these first two definitions (Rothlin and Werder, 2009; Stock, 2015), bore-out seems to be composed of three main components. They derive from boredom at work and job meaning with a *lack* or a *crisis* related to work environment, but studies in this field have recently added a social dimension to the definition of bore-out (Hosy and Bourion, 2017; Özsungur, 2020a,b). For Özsungur (2020a), bore-out is the consequence of employees' experiences, interactions, communication, and perception of work. The results of several studies confirmed that negative relationships with a manager or mobbing at work increases the level of employee bore-out (Özsungur, 2020a,b). However, this social dimension is also integrated in bore-out by social comparison. Social norms at work creates shame and guilt leading bored employees to experience bore-out (Bourion and Trebucq, 2011). In this case, they cannot express their ill-being or their boredom because of shame (Brühlmann, 2015) and they feel guilty for complaining about not having anything to do (Rengade, 2016; Chapelle, 2018; Fiedler and Nauta, 2020). According to the research, bore-out also has a fourth dimension that is guilt (Bataille, 2016; Chapelle, 2018) or even shame felt by employees (Bourion and Trebucq, 2011) concerning their own work in comparison with that of their colleagues.

### Variables Related to Bore-Out

Bore-out is a recently defined concept. Based on these conceptual frameworks (Rothlin and Werder, 2009; Stock, 2015), some studies have investigated the link between bore-out and several other variables. To our knowledge, no longitudinal and experimental study has been conducted to explore the consequences of bore-out. However, studies have shown that bore-out has several correlates at different levels (Chapelle, 2016): professional strain, reduced work performance, non-productive behaviors, absenteeism, or psychopathologies (e.g., depression, anxiety). Bore-out could decrease employees' self-esteem (Hosy and Bourion, 2017) and is positively associated with depression and stress (Özsungur, 2020b). Additionally, in the service industry, bore-out seems to negatively influence employees' satisfaction (e.g., career, life, and job), but this effect is greater for older employees (i.e., Gen-X) born before 1980 (Abubakar, 2019). In a study looking specifically at cabin crew, Karatepe and Kim (2020) showed that bore-out negatively affects their ability to identify passengers' needs, be engaged at work and service-oriented organizational citizenship behaviors.

Stock (2015, 2016) investigated each component of bore-out (i.e., *job boredom, crisis of growth*, and *crisis of meaning*) among various personal services in a business-to-consumer (e.g., retailing, consulting, insurances). The *crisis of growth* and the *crisis of meaning* at work are negatively associated with employees' innovative work behavior and positively with *job boredom* (Stock, 2015). In a second study with frontline employees, Stock (2016) showed that all three dimensions of bore-out negatively affected customer-oriented behavior.

Although several studies have investigated the consequences of bore-out during the last decade, the material used to measure bore-out is not always the same. Some studies have used interviews (Bourion and Trebucq, 2011; Hosy and Bourion, 2017), while others have used scales (Stock, 2015, 2016; Abubakar, 2019; Karatepe and Kim, 2020; Özsungur, 2020a).

### Measures of Bore-Out

So far, three tools (two scales and one multi-scale construct) have been developed to measure bore-out. The first was developed by the Swiss consultants Rothlin and Werder (2009) and consisted of ten yes/no questions (e.g., “Do you feel helpless or bored?”). The more yes answers they recorded, the higher the employee bore-out was. However, this scale has not been validated or reused in other studies. The second scale was developed by Spanish researcher Noriega (2014) based on Rothlin and Werder's definition. Noriega's scale includes 18 items scored from 0 = never to 4 = always. Each item aims to measure one of the three components: *boredom* (e.g., “I spend all day waiting for it to end”), *lack of interest* (e.g., “I find my work insignificant”), *lack of challenge* (e.g., “My boss delegates insignificant tasks to me”). Unfortunately, reliability is low (0.71 > α > 0.46), no confirmatory analysis has been performed and the scale's validity has not been thoroughly examined.

The third and most used measure of bore-out was proposed by Stock (2015) and is a combination of three different scales. According to his own definition of bore-out, Stock (2015, 2016) used 11 items from these differently validated scales. In order to measure the *crisis of meaning*, a reduced scale (4 items) from Schnell (2010) was used after changing the word “life” to “work” (e.g., “My work seems meaningless”). Three items measuring *job boredom (e.g., “In my job I feel bored”)* were taken from the scale originally developed by Fisher (1993). Finally, the *crisis of growth* was measured with a reversed version of the scale developed by Bakker et al. (2010; e.g., “*I learn new things in my work*”). The scale is calibrated by the 7-point Likert scale (Stock, 2015), although Abubakar (2019) used a 5-point Likert scale.

Although these measures were used in several studies, these scales only included the three components of bore-out as defined by Rothlin and Werder (2009) or Stock (2015). However, studies in this field suggest also that social comparison, through the feeling of shame and guilt, is a component of bore-out (Bataille, 2016; Rengade, 2016; Hosy and Bourion, 2017; Fiedler and Nauta, 2020).

### Aim of the study

Based on the current observation that the notion of bore-out is gaining more attention and building on the pioneering work of Rothlin and Werder (2009) and Stock (2015), the aim of this study was to go a step further by introducing new components (e.g., shame and guilt) that have emerged from recent studies (Rengade, 2016; Chapelle, 2018; Fiedler and Nauta, 2020) and examine the psychometric properties of the resulting scale in a sample of French-speaking employees. In addition, we want to validate our construct for all French-speaking employees no matter their gender or age. The perception of bore-out seems different between young and older employees (Hosy and Bourion, 2017; Abubakar, 2019). We need to confirm the factorial structure of our scale across generations. In the same way, employee's gender is an important issue for the validity of a scale (Hyde, 2005). This concern is important in the current study because the samples for most studies in the field had an average of 40% of women (Stock, 2015, 2016; Özsungur, 2020a,b,c) which is lower than the general population in France (48% of women; INSEE, 2020). Our scale needs to be tested and adapted for French-speaking men and women employees. In order to do this, we examined (a) creation of our scale, (b) its construct validity (through exploratory and confirmatory analyses), (c) its construct validity by gender and generation (born before 1980, or in 1980 or after; Abubakar, 2019), (d) the reliability and (e) the relations with other variables. The links with other variables were observed between our measure and potential correlates of bore-out (i.e., self-esteem, depression, perceived helplessness, and perceived self-efficacy). To go further than previous studies (Özsungur, 2020a,b,c), we used the two subscales of professional stress: perceived helplessness and perceived self-efficacy. In line with the literature (Hosy and Bourion, 2017; Özsungur, 2020a,b)(Hosy and Bourion, 2017; Özsungur, 2020a,b), we expected to find a negative link between bore-out and self-esteem, and positive links between bore-out and depression, perceived helplessness and perceived self-efficacy.

## Methods

### Participants

A total of 741 French-speaking workers participated in the study. We kept only completed questionnaires (*N* = 507) for our analysis. There were 347 women (68%) and 160 men (32%). Our participants were aged from 22 to 66 years (mean age = 43.8; *SD* = 9.6), with 324 (44%) of them born before 1980 (Gen-X). A majority of participants were graduates (*n* = 329), 143 participants were undergraduates, and 35 had a high school education. The participants were working in companies which employed 10 workers or less (*n* = 54), between 11 and 249 employees (*n* = 156), between 250 and 4999 employees (*n* = 183), or more than 5,000 employees (*n* = 100) or were self-employed (*n* = 14).

### Procedure

Data were collected from French-speaking employees who had been informed about the research through social media, websites, or by word of mouth. After the study had been presented and the participants had given their informed consent to take part voluntarily and anonymously, in accordance with the GDPR's regulations, they completed the survey on a specific secure online platform (LimeSurvey). The items including in our new scale have been randomly presented.

### Creation of Work Bore-Out Scale (WBOS)

The Work Bore-Out Scale (*WBOS*) was developed in French and based on a theoretical framework as well as previous measures. Theoretical and empirical studies (Rothlin and Werder, 2009; Noriega, 2014; Stock, 2015, 2016) seem to indicate several bore-out components. Recent studies (Chapelle, 2016) have added negative feelings regarding social comparison (e.g., guilt, shame) to bore-out components. Two semi-structured interviews were conducted, as recommended by Churchill (1979), with employees on the subject of bore-out. These two employees were interviewed about their work situation, the characteristics of a bore-out situation, their difficulties and their feelings related to bore-out. The information collected has been coded and classified into two main categories: risk factors of bore-out and compensatory strategies. The list of risk factors has been compared to items from literature review (e.g., Noriega, 2014). After this qualitative exploratory phase, 20 items were created or adapted covering these components. Items were rated on a scale from 1 = never to 5 = often. Three of them were reversed.

### Measures

In addition to our French work bore-out scale and sociodemographic information, three other questionnaires were included to measure the relation with other variables.

#### Self-Esteem

Self-esteem was assessed with *Rosenberg's Self-Esteem Scale (RSE;* Rosenberg, 1965; Vallieres and Vallerand, 1990 for French version). The 10 items of the *RSE* are scored on a scale from 1 = totally disagree to 4 = totally agree. The global score is obtained by summing the 10 item scores. Cronbach's alpha for the current sample (0.87) was close to that described by Vallieres and Vallerand (1990).

#### Depression

Depression was assessed with the short form of the *Beck Depression Inventory (BDI;* Beck, 1961; Bourque and Beaudette, 1982 for French version). The *BDI* is a 13-item, self-report questionnaire. Items are scored on a scale from 0 to 3. The depression score is obtained by summing the 13 item scores. Cronbach's alpha for the current sample was 0.89, which was similar to that found by Bourque and Beaudette (1982).

#### Perceived Helplessness and Perceived Self-Efficacy

*Perceived helplessness* was measured via the *perceived helplessness scale* of the *Perceived Stress Scale-10 items (PSS 10*; Cohen et al., 1983), validated in French by Bellinghausen et al. (2009). The six items are scored on a scale from 1 = never to 5 = often. Cronbach's alpha for the current sample was 0.88, similar to that found by Bourque and Beaudette (1982).

*Perceived self-efficacy* was measured via the *perceived self-efficacy scale* also included in the *Perceived Stress Scale-10 items (PSS 10*; Cohen et al., 1983; adapted in French by Bellinghausen et al., 2009). The four items are scored on a scale from 1 = never to 5 = often. For this scale, Cronbach's alpha for the current sample was 0.83, close to that described by Bourque and Beaudette (1982).

### Data Analyses

Validation of the questionnaire was conducted according to the standards and guidelines of the American Psychological Association (AERA, 2014). We examined the internal and external structure of the questionnaire with the Rstudio package. For the purpose of factor analyses, the sample was split into two subsamples in order to compute principal component analyses (PCA) and confirmatory factor analysis (CFA) on two different samples (Pohlmann, 2004).

We started by factor-analyzing the 20 items reflecting bore-out on the first subsample. With regard to the internal structure of the questionnaire, principal component analysis (PCA) was conducted after checking the sufficiency of item correlations with Bartlett's test (*p* < 0.05) and the Kaiser-Meyer-Olkin (KMO) test (close to 1). Promax rotation was used considering potential links between our factors. The number of components was based on the Kaiser-Guttman Rule (eigenvalues > 1). Items were excluded if they had factor loadings lower than |0.30| or factor loadings higher than |0.30| on more than one factor (e.g., Tabachnick et al., 2019), and also when their uniqueness was >0.60 (Broc et al., 2016). Reliability was estimated with Cronbach's alpha and McDonal's omega, a coefficient ≥0.80 denoting good internal consistency (Dima, 2018). This analysis was performed using the Psych package (Revelle, 2019).

Confirmatory factor analyses (CFAs) were performed, from a model observed in PCA, on the second subsample. In order to take account of the ordinal nature of the data, diagonally weighted least squares with a polychoric correlation matrix were used. Several goodness-of-fit measures were used to determine the acceptability of the models. The analyses used the robust maximum likelihood estimator, which takes account of non-normal data distribution. This analysis was performed using the Lavaan package (Rosseel, 2012). The Root Mean Square Error of Approximation (RMSEA), the Standardized Root Mean Square Residual (SRMR), the Comparative Fit Index (CFI) and the Tucker-Lewis Index (TLI) were used. Values close to or >0.95 for CFI and TLI, and < 0.08 for SRMR are acceptable (Hu and Bentler, 1999) and RMSEA should be < 0.08 (Steiger, 2007).

Moreover, we analyzed invariance across gender and age groups using a series of multiple-group confirmatory factor analysis models with progressively more stringent constraints. Four models were performed to test measurement and structural invariance: configural, metric, scalar, strict (van de Schoot et al., 2012). Configural invariance was specified to have the same pattern of free and fixed parameters across groups, without any equality constraints. This enabled us to examine whether the same items measured the same constructs across groups. In metric invariance, only the factor loadings were constrained to be equal across groups. This model implied that the same latent variables were measured across groups. Scalar invariance was tested by specifying factor loadings and thresholds to be invariant across groups. Strict invariance had an additional constraint that uniquenesses were invariant across groups. A more constrained model was rejected when (a) the Chi^2^ difference test had a probability lower than 0.05 (Byrne and van de Vijver, 2010), (b) the Δ CFI showed a decrease of more than 0.010 (Cheung and Rensvold, 2002) and (c) the Δ RMSEA showed an increase of more than 0.015 (Chen, 2007). As French and Finch (2006) recommend the use of the Chi^2^ difference test criterion in multiple-group confirmatory factor analysis with a large number of factors, this criterion was privileged. This analysis was performed using semTools and the Lavaan package (Enders, 2008; Rosseel, 2012). Each model was tested with weighted least square mean and variance-adjusted estimation.

With regard to the relation between WBOS and other variables, the specificity of bore-out was investigated by examining its correlations with depression, self-esteem, perceived helplessness and perceived self-efficacy. According to Cohen (1988) conventional criteria, effect size was deemed small when close to 0.30, moderate when close to 0.40 and large when close to 0.50 or greater. We used the conservative Bonferroni correction method with its correlations.

## Results

### Factor Structure and Reliability

Two principal component analyses with oblique rotation (promax) were conducted on the 20 items on a first subsample (*n* = 253). The first model, including a pool of 20 items, explained 64% of the variance, with four factors (eigenvalue > 1). The KMO index of 0.93 indicated good sampling adequacy and the Bartlett sphericity test was significant (χ^2^ = 2829.8, *p* < 0.001). The factor loading revealed several items that presented complex saturations. Following our first PCA, 5 items were removed: 2 with evident cross-loadings across two factors (> |0.30|) and 3 with high uniqueness (>0.60). A second PCA, including 15 items, was run and converged toward a four-factor model explaining 73% of the variance. In this second version, the KMO index was 0.90 and the Bartlett sphericity test was significant (χ^2^ = 2121, *p* < 0.001). All factor loadings are presented in Table 1. In addition, the reliability of our scale was good. The internal consistencies of the four subscales were high. Cronbach's alphas for all subscales exceeded 0.80 as shown in Table 1. Based on the items' meanings, the first factor was labelled *insufficient workload*, the second *under-stimulation*, the third *work-related guilt* and the fourth *incompatibility of personal work values*. The complete list of conserved items is available in the Appendix in Supplementary Material.

**Table 1:** Descriptive statistics, loadings, uniqueness, internal consistency and eigenvalues.

						**Factors**			
	***M***	***SD***	***Min***	***Max***	**1**	**2**	**3**	**4**	**μ**
1-Insufficient workload	1.6	0.8	1	5					
Item 1	1.6	1.0	1	5	**0.86**				0.24
Item 2	1.9	1.1	1	5	**0.94**				0.26
Item 3	1.6	1.2	1	5	**0.87**				0.34
Item 4	1.4	0.9	1	5	**0.65**				0.44
Item 5	1.6	1.5	1	5	**0.78**				0.22
2-Understimulation	2.6	1.1	1	5					
Item 6	3.0	0.9	1	5		**0.76**			0.38
Item 7	2.3	1.2	1	5		**0.73**			0.39
Item 8 (R)	2.8	1.3	1	5		**0.95**			0.23
Item 9	2.1	1.3	1	5		**0.81**			0.30
Item 10 (R)	2.7	1.3	1	5		**0.91**			0.19
3-Work-related guilt	1.9	1.0	1	5					
Item 11	2.1	1.3	1	5			**0.99**		0.21
Item 12	1.8	1.1	1	5			**0.77**		0.27
Item 13	1.8	1.2	1	5			**0.70**		0.32
4- Incompatibility of personal work values	3.1	1.4	1	5					
Item 14	3.1	1.3	1	5				**0.91**	0.16
Item 15	3.1	1.5	1	5				**0.92**	0.16
Eigenvalues					1.97	6.40	1.00	1.53	
% of variance explained					24	23	14	12	
Cronbach's alphas					0.89	0.88	0.81	0.81	
95%CI alpha					0.87–0.90	0.86–0.90	0.78–0.84	0.77–0.84	
McDonal's omega					0.90	0.88	0.78	0.79	

*(R) reversed items. Significant factors loadings are indicated in bold. μ = uniqueness*.

The correlations between the four factors were 0.34 (factor 1-factor 2), 0.40 (factor 1-factor 3), 0.03 (factor 1-factor 4), 0.52 (factor 2-factor 3), 0.19 (factor 2-factor 4) and 0.27 (factor 3-factor 4). The correlation between factor 1 and factor 4 was not significant.

The factor model extracted by the PCA was cross validated using confirmatory factor analyses (CFA) in a second subsample (*n* = 254). All the estimated factor loadings found in the CFA were significant at *p* < 0.001. The indices examined to assess correspondence between the theoretical and observed models suggested a good fit of our models compared with the expected values: χ^2^ (84) = 212.7, CFI = 0.98, TLI = 0.97, RMSEA = 0.078 [0.065–0.091], SRMR = 0.063. In view of the lack of correlations between *incompatibility of personal work values* and the other factors, a second CFA was run without this factor. However, the results showed a lower fit than with the four-factor model: χ^2^ (62) = 215.9, CFI = 0.97, TLI = 0.96, RMSEA = 0.099 [0.085–0.114], SRMR = 0.070. The fourth factor was retained for further analysis.

To confirm the factor structure across genders, in the first step, two CFAs with four factors were conducted on samples of men (*n* = 160) and women (*n* = 347). These two models presented acceptable values: χ^2^ (84) = 134.2, CFI = 0.99, RMSEA = 0.061 [0.041–0.080], TLI = 0.98 and SRMR = 0.064 for men; χ^2^ (84) = 304.6, CFI = 0.97, RMSEA = 0.087 [0.077–0.098], TLI = 0.97 and SRMR = 0.066 for women. A series of multiple-group confirmatory factor analysis models were used in the second step. The results are presented in Table 2. The configural invariance model showed acceptable criteria and could be used as the baseline model. The metric invariance model demonstrated acceptable criteria. The scalar invariance model had a good fit and was not rejected, as was also the case for the strict invariance model. All loadings, thresholds, and uniquenesses proved to be invariant across gender.

**Table 2:** Multiple-group confirmatory factor analysis models for measurement and structure invariance across gender.

	**χ^2^**	***df***	**RMSEA [90CI%] (Δ)**	**CFI (Δ)**	***p***
Configural invariance	276.10	168	0.050 [0.04–0.061]	0.996	
Metric invariance	308.04	179	0.053 [0.043–0.063] (0.003)	0.996 (0.000)	0.62
Scalar invariance	356.61	219	0.050 [0.040–0.059] (0.003)	0.995 (0.001)	0.54
Strict invariance	356.61	234	0.046 [0.036–0.055] (0.004)	0.996 (0.001)	1

As in the invariance analysis for genders, in the first step, two CFAs with four factors were conducted for Gen-X (born before 1980, *n* = 324) and Gen-Y (born in 1980 or after, *n* = 183) samples. These models presented acceptable values for Gen-X (χ^2^ (84) = 248.7, CFI = 0.98, RMSEA = 0.078 [0.067–0.089], TLI = 0.97 and SRMR = 0.059) and Gen-Y (χ^2^ (84) = 191, CFI = 0.97, RMSEA = 0.084 [0.068–0.099], TLI = 0.97 and SRMR = 0.074). In the second step, multiple-group confirmatory factor analysis models were run. The results are presented in Table 3. The configural invariance model could be used as the baseline model, showing acceptable criteria. The metric and scalar invariance models had good fits and were not rejected, and the same was true of the strict invariance model. All loadings, thresholds, and uniquenesses proved to be invariant across generations.

**Table 3:** Multiple-group confirmatory factor analysis models for measurement and structure invariance across generations.

	**χ^2^**	***df***	**RMSEA [90CI%] (Δ)**	**CFI (Δ)**	***p***
Configural invariance	282.2	168	0.052 [0.041–0.062]	0.996	
Metric invariance	298.5	179	0.051 [0.041–0.061] (0.001)	0.996 (0.000)	0.83
Scalar invariance	362.9	220	0.051 [0.041–0.060] (0.000)	0.995 (0.001)	0.07
Strict invariance	*362.9*	235	0.046 [0.037–0.056] (0.005)	0.996 (0.001)	1

### Relation With Other Variables

The correlations between the four subscales of the work bore-out (*WBOS*), *RSE, BDI, Perceived helplessness and Perceived self-efficacy* questionnaires are presented in Table 4. Significant relations existed between work bore-out (*WBOS*), self-esteem (*RSE*), depression (*BDI*), *perceived helplessness* and *perceived self-efficacy*, but not for all subscales of *WBOS*. Except for *incompatibility of personal work values*, the subscale scores of *WBOS* increased as the score of *RSE* decreased (−0.21 < ρ < −0.46, *p* < 0.001). By contrast, as the *WBOS* subscale scores increased, so too did the *BDI* score (0.28 < ρ <0.51, *p* < 0.001). Our results showed positive and significant links between the *under-stimulation* and *work-related guilt* subscales and *perceived helplessness* (0.19 < ρ < 0.33, *p* < 0.001). Finally, we observed significant correlations between almost all subscales of *WBOS* and *perceived self-efficacy* (0.26 < ρ <0.58, *p* < 0.001). The *incompatibility of personal work values* factor did not correlate with any of these scales and the *insufficient workload* factor did not correlate with *perceived helplessness*.

**Table 4:** Bivariate correlations.

	**Bore-out (***WBOS***)**			
	**Under-loaded**	**Under-stimulated**	**Guilty of work life**	**Inadequacy of personal work values**
Self-esteem (*RSE*)	−0.21^***^	−0.39^***^	−0.46^***^	−0.03
Depression (*BDI*)	0.28^***^	0.54^***^	0.39^***^	0.03
Perceived Helplessness	−0.02	0.33^***^	0.19^***^	−0.03
PerceivedSelf-Efficacy	0.26^***^	0.58^***^	0.35^***^	−0.03

*** *adjusted-p < 0.001*.

## Discussion

Building on previous studies in the field and aiming to overcome some limitations (e.g., scales' psychometric quality, lack of bore-out components), this study sought to develop a new scale to measure bore-out at work. The current findings provide preliminary evidence that the resulting scale, the Work Bore-Out Scale (WBOS), presents adequate psychometric properties: its subscales scores are reliable, its factor structure has theoretical meaning and is invariant across genders and generations, and subscales scores alike correlate in the expected way with external variables.

Although items were initially developed to cover the main concepts including in bore-out's definitions (e.g., boredom, lack of challenge, lack of growth, social comparison), the analyses suggested that bore-out is best represented by different dimensions. The four factors that emerged from our analyses were labelled *insufficient workload, under-stimulation, work-related guilt* and *incompatibility of personal work values*.

The first factor, labelled *insufficient workload*, represents a lack of work. Workers with a high score on this factor “spend hours ‘not knowing what to do,”' “do not have enough work,” or do other things than work to pass their time (e.g., “managing personal affairs”, “taking breaks”). This dimension of bore-out represents the first side of boredom, stemming from having nothing to do (Rothlin and Werder, 2009; Stock, 2015).

The second factor, *under-stimulation*, represents the second side of boredom, due to taking part in monotonous, boring, or meaningless activities (Noriega, 2014). The tasks of workers who score highly on this factor are “insignificant and uninteresting.” In addition, this *under-stimulation* is also reflected in the feeling that their full potential is not being valued (e.g., “I feel that my skills and knowledge are not being used to their full potential”).

The third factor, *work-related guilt*, is not directly represented in other bore-out measures (Noriega, 2014; Stock, 2015). However, studies have shown that guilt or shame regarding one's work situation is a key feeling that characterizes bore-out (Baumann, 2016; Chapelle, 2018). This factor (*work-related guilt*) describes how employees feel “guilty for not working enough” or are “ashamed by their workflow.”

The fourth factor, labelled *incompatibility of personal work values*, corresponds to the cognitive dissonance an employee might have regarding their work situation (e.g., “Having little work is not the way I would like to work”). When an employee has worked less than they expected, an imbalance could appear between her work values and her work reality. Although several studies have presented this imbalance as being a part of bore-out (Bourion and Trebucq, 2011), this factor has not appeared in previous scales (e.g., Stock, 2015).

As expected, our study shows negative relations between bore-out and self-esteem as well as positive relations between bore-out and depression, perceived helplessness and perceived self-efficacy. These relations are evident at the construct level but also at the factor level (with varying strength), except for *incompatibility of personal work values* which does not correlate with any of the external variables included here. We will return to this result later. The other three subscales of bore-out are negatively linked to self-esteem: if an employee is under-worked, under-stimulated, or feels guilty, they will have little confidence in their skills and low self-esteem, as already suggested in a previous study (Hosy and Bourion, 2017). These factors also correlate with depression as expected (Özsungur, 2020b). Employees suffering bore-out feel guilt at having nothing to do, meaninglessness, or anxiety (Özsungur, 2020b), leading them to depressed thoughts (Chapelle, 2018). Finally, *perceived helplessness* and *perceived self-efficacy* are included in this study because of their association with bore-out (Özsungur, 2020b). By contrast with Özsungur (2020a,b), our results did not show significant links between all subscales of WBOS and the two components of perceived stress *(perceived helplessness* and *perceived self-efficacy). Under-stimulation* and *work-related guilt* are positively linked with low self-efficacy and high helplessness. However, the results show that the *insufficient workload* factor does not correlate with the score for *perceived helplessness*. Hewitt et al. (1992) define *perceived helplessness* as a distress component included in negative affective reactions such as anger, being upset, and nervousness. However, our results seem more congruent with Bellinghausen et al. (2009). For them, *perceived helplessness* means being emotionally and cognitively overwhelmed. This implies that the feeling of being under-worked is not enough to experience helplessness at work. There is not a complete overlap between the concepts under consideration: work bore-out is not just low self-esteem and it is not just depression, perceived helplessness, or perceived self-efficacy.

The fourth factor (*incompatibility of personal work values*) did not show the expected links. This factor concerns the perceived gap between the demands of work and the employee's expectations (Bourion and Trebucq, 2011). Where such a gap is perceived to exist, the resulting cognitive dissonance may create an imbalance (Festinger, 1962). Although this perceived gap could be linked to depressed thinking or stressors (Chapelle, 2018), our results did not find a significant correlation with other variables.

Overall, except for the *incompatibility of personal work values* factor, the results correspond to our expectations based on previous studies on the consequences of bore-out. Our results complement the viewpoint of Rothlin and Werder (2009) and Stock (2015). To be under-worked is not enough to suffer from bore-out. Although boredom, expressed as ‘insufficient workload', appears as the first factor of bore-out (Rothlin and Werder, 2009), our study shows the need to take account of employees' stimulation as well as of their feelings about their work. Bore-out does not arise purely from boredom at work (insufficient workload and under-stimulation): negative feelings relating to this situation are components too. Our results show that guilt about workload is an essential component of bore-out as suggested by Hosy and Bourion (2017), Rengade (2016) or even Bourion and Trebucq (2011). The feeling of guilt for their own work leads employees to have a negative perception of their social identity which is a part of bore-out (Özsungur, 2020a,b). Conversely, the fourth factor (*incompatibility of personal work values*) is unexpected in view of the theoretical components of bore-out. Although the four-factor model presents a good fit, the incompatibility between personal values and work reality should perhaps be better viewed as a distinct construct. To confirm these preliminary results, further investigations are necessary.

### Limitations and Further Research

In spite of its strengths, several limitations have to be acknowledged, which open interesting avenues for future research. The first possible limitation is the lack of convergent validity. Despite the good fit of our model and the presence of the relations with other variables that were expected in light of our theoretical approach, the model's convergent validity was not directly tested. Further study could be done to investigate the link between *WBOS* and the boredom scale (Reijseger et al., 2013) or the scales used by Stock (2015).

Secondly, the study includes only French employees. The perception of bore-out could be different in other cultures, as is the case for burn-out (Schaufeli et al., 2009). The results provide preliminary evidence in favor of the validity of our questionnaire, but further cross-cultural research has to be done to confirm the factor structure of WBOS in other countries/languages.

The third limitation is the participation rate between men and women. The rate of women employees in our sample was higher than in French general working population (68% vs. 48%; INSEE, 2020). Even if this rate did not impact the factorial structure of our scale which was confirmed by invariance of the measurement for men and women, the relations with other variables could be impacted by this rate. Further studies need to be made to confirm our findings in samples with different rates.

The fourth limitation is the type of material used in our study. All measures are based on self-report questionnaires (e.g., *BDI, RSE*). In order to strengthen the predicted validity of *WBOS*, several objective variables (e.g., turnover, absenteeism) could be used with a longitudinal design study. Moreover, our study used a variable-centered approach; a person-centered approach would make it possible to observe bore-out profiles and their prevalence in further research. This approach could be linked to clinical interviews. Both person-centered approach and clinical interviews could be used to confirm our scale and identify specific clinical implications of bore-out in the same way.

## Conclusion

To conclude, the current study aimed to develop a validated tool to measure bore-out in the workplace. The content of WBOS was based on a theoretical framework (Hobfoll, 1989, 2001; Rothlin and Werder, 2009; Stock, 2015, 2016) and built on previous studies in this field (Bourion and Trebucq, 2011; Abubakar, 2019; Karatepe and Kim, 2020; Özsungur, 2020a,b). Our findings led to the development of a four-factor model and complemented existing definitions of bore-out (Rothlin and Werder, 2009; Stock, 2015; Baumann, 2016; Chapelle, 2016, 2018). This instrument can be used by French employees of all types as a first step to become aware of their level of bore-out and consequently lead them to act for their well-being at work. Moreover, quality of work life programs can be tailored based on the WBOS results. These programs could identify and reduce the predictors of bore-out. Özsungur (2020a) suggested a training manager but colleagues could act too. Further investigation is needed to confirm the link between potential consequences of bore-out (e.g., depression, turnover, anxiety) and the beneficial effects to reduce bore-out (e.g., social interaction, performance).

## Data Availability Statement

The datasets presented in this study can be found in online repositories. The names of the repository/repositories and accession number(s) can be found below: https://osf.io/r9xw3/?view_only=f1b6b866d5784dcbad76d4a42ee72f40.

## Ethics Statement

Ethical review and approval was not required for the study on human participants in accordance with the local legislation and institutional requirements. The patients/participants provided their written informed consent to participate in this study.

## Author Contributions

MG devised the theoretical framework of the bore-out in the framework of a discussion with MM. The authors worked together to operationalize it, design the study. MG and CP collect the data. CP analyzed the data and wrote the Methods and Results section. MG and CP wrote the Introduction and Discussion sections. All authors commented on, proofread and approved the final version of the paper.

## Conflict of Interest

The authors declare that the research was conducted in the absence of any commercial or financial relationships that could be construed as a potential conflict of interest.

## Publisher's Note

All claims expressed in this article are solely those of the authors and do not necessarily represent those of their affiliated organizations, or those of the publisher, the editors and the reviewers. Any product that may be evaluated in this article, or claim that may be made by its manufacturer, is not guaranteed or endorsed by the publisher.
