# Supplementary material for: Creation and Validation of the First French Scale for Measuring Bore-Out in the Workplace
Source: Front Psychol. 2021 Jul 23;12:697972. doi: 10.3389/fpsyg.2021.697972 (PMC8343021; doi:10.3389/fpsyg.2021.697972)
Supplement: Supplementary file 1 [file Data_Sheet_1.PDF]

List of items
Factor 1: Insufficient workload
Item 1: I can spend hours « not knowing what to do »<br>*Je peux passer des heures à "ne pas savoir quoi faire"*
Item 2: I very often manage personal affairs at work to pass the time<br>*Je gère très souvent des affaires personnelles au travail pour combler le temps*
Item 3: At work, I spend a lot of time chatting and taking breaks, because I don't have a lot to do<br>*Au travail, je passe beaucoup de temps à discuter, à prendre des pauses, faute de choses à faire*
Item 4: I spend my time asking my superiors for work, without success<br>*Je passe mon temps à demander du travail à ma hiérarchie, sans succès*
Item 5: I don't have enough work<br>*Je n'ai pas assez de travail*
Factor 2: Understimulation
Item 6: I feel that my skills and knowledge are not being used to their full potential<br>*J'ai le sentiment que mes compétences et connaissances ne sont pas exploitées à leur juste valeur*
Item 7: At work, I often have to carry out completely meaningless tasks<br>*Au travail, je dois souvent réaliser des missions complètement dénuées de sens*
Item 8: My tasks are stimulating with regard to my professional skills (R)<br>*Les tâches qui me sont confiées sont stimulantes au regard de mes compétences professionnelles (R)*
Item 9: My work is insignificant and uninteresting<br>*Le travail que l'on me donne est insignifiant et inintéressant*
Item 10: I am motivated by my working tasks (R)<br>*Les tâches qui me sont confiées me motivent (R)*
Factor 3: Work-related guilt
Item 11: I very often feel guilty for not working enough<br>*Je me sens très souvent coupable de ne pas assez travailler*
Item 11: I have lost all confidence in myself and my skills because I didn't have enough work<br>*J'ai perdu toute confiance en moi et en mes compétences à force de ne pas assez travailler*
Item 12: I am very often ashamed to talk about my work rate<br>*J'ai très souvent honte de parler de mon rythme de travail*
Factor 4: Incompatibility of personal work values
Item 13: Having little work is not the way I would like to work<br>*Le fait d'avoir peu de travail ne correspond pas à la façon dont je souhaiterais travailler*
Item 14: Having little work is at odds with my idea of the working world<br>*Le fait d'avoir peu de travail contraste avec l'idée que je me fais du monde du travail*
Note. (R) reversed items. Original French items are presented in *italic*.
